# Genetic variants in *IL17A* and serum levels of IL-17A are associated with COPD related to tobacco smoking and biomass burning

**DOI:** 10.1038/s41598-020-57606-6

**Published:** 2020-01-21

**Authors:** Marco A. Ponce-Gallegos, Gloria Pérez-Rubio, Enrique Ambrocio-Ortiz, Neftali Partida-Zavala, Rafael Hernández-Zenteno, Fernando Flores-Trujillo, Leonor García-Gómez, Andrea Hernández-Pérez, Alejandra Ramírez-Venegas, Ramcés Falfán-Valencia

**Affiliations:** 1 HLA Laboratory, Instituto Nacional de Enfermedades Respiratorias Ismael Cosío Villegas, Mexico City, 14080 Mexico; 2 Tobacco Smoking and COPD Research Department, Instituto Nacional de Enfermedades Respiratorias Ismael Cosío Villegas, Mexico City, 14080 Mexico

**Keywords:** Disease genetics, Genetics research

## Abstract

IL-17A is an important pro-inflammatory cytokine involved in the inflammatory response in chronic obstructive pulmonary disease (COPD). To evaluate the role played by single nucleotide polymorphisms of *IL17A* and protein levels in susceptibility to COPD, 1,807 subjects were included in a case-control study; 436 had COPD related to tobacco smoking (COPD-S) and 190 had COPD related to biomass burning (COPD-BB). Six hundred fifty-seven smokers without COPD (SWOC) and 183 biomass burning-exposed subjects (BBES) served as the respective control groups. The CC genotype and C allele of rs8193036 were associated with COPD (COPD-S *vs*. SWOC: p < 0.05; OR = 3.01, and OR = 1.28, respectively), as well as a recessive model (p < 0.01; OR = 2.91). Significant differences in serum levels were identified between COPD-S *vs*. SWOC, COPD-S *vs*. COPD-BB, and SWOC *vs*. BBES (p < 0.01). By comparing genotypes in the COPD-BB group TT *vs*. CC and TC *vs*. CC (p < 0.05), we found lower levels for the CC genotype. Logistic regression analysis by co-variables was performed, keeping the associations between COPD-S *vs*. SWOC with both polymorphisms evaluated (p < 0.05), as well as in COPD-BB *vs*. BBES but with a reduced risk of exacerbation (p < 0.05). In conclusion, polymorphisms in *IL17A* are associated with COPD. Serum levels of IL-17A were higher in smokers with and without COPD.

## Introduction

According to the Global Initiative for Chronic Obstructive Lung Diseases (GOLD) guidelines, chronic obstructive pulmonary disease (COPD) is a common, preventable and treatable disease that is distinguished by persistent respiratory symptoms and airflow limitation, which is due to airway abnormalities usually caused by significant exposure to noxious particles or gases, with tobacco smoking and biomass burning indoors being the major risk factors^1^.

It is estimated that the prevalence in developed countries oscillates in 3 to 6% of subjects older than 50 years^2^. The World Health Organization (WHO)^3^ states that in 2030, COPD will rank fourth among causes of death worldwide. In addition, in México, this disease is ranked fourth in terms of morbimortality^2^.

Despite being one of the most prevalent diseases in developed countries, the pathogenesis and factors involved in COPD have not been fully elucidated. Nevertheless, genetic associations have expanded our vision and have elucidated important mechanisms that participate in COPD. An imbalance in protease – antiprotease (α-1 antitrypsin deficiency) has been reported as one of the most important genetic factors associated with the presence of this entity^4^. Furthermore, single nucleotide polymorphisms (SNPs) in proinflammatory genes, such as *TNF*, *CXCR2*, *CXCL8*, *ADAM19*, and *IL6R*, have been described as an important risk factor for COPD and its severity^5–7^.

On the other hand, the Th17 profile has been studied widely in autoimmune diseases and has been described as a risk factor. For example, there are studies of SNPs in *IL17A* associated with rheumatoid arthritis and systemic lupus erythematosus^8–11^, but there are not enough studies in COPD, despite the pathophysiological role of IL17 and Th17 cells. Since 2003, Agustí and coworkers^12^ hypothesized that one of the possible pathogenic mechanisms in COPD is the existence of self-perpetuating inflammation, similar to other autoimmune diseases. This idea is supported by studies where autoantibodies have been identified in these patients, as well as previous reports that describe ongoing inflammation in COPD patients who do not currently smoke^13–16^. Several studies have demonstrated an increasing concentration of interleukin-17A (IL-17A) in central and distal airway lung tissue in these patients^17,18^. Additionally, Th17 cells are increased in the peripheral blood in patients with COPD^19^.

Th17 cells participate in immunity against extracellular bacteria and recruit neutrophils to the inflammation zone. Therefore, in COPD patients, these cells could be an important factor of the onset of exacerbations, which are defined as worsening of the daily symptomatology that requires modifications in the treatment^1,20^. Therefore, due to the significant mortality and the impact on the quality of life caused by exacerbations in this kind of patient, it is necessary to investigate genetic susceptibility to COPD and COPD exacerbations.

The aim of our study was to identify *IL17A* SNPs that confer risk to COPD related to biomass burning smoke exposure and tobacco smoking and are associated with frequent exacerbations.

## Materials and Methods

### Study population

#### Case and control groups

A total of 1,807 subjects were included in the case-control study. These subjects attended the COPD and smoking cessation support clinics, both part of the Department of Smoking and COPD Research Department of the Instituto Nacional de Enfermedades Respiratorias Ismael Cosio Villegas (INER), Mexico. The sample included 436 patients with a diagnosis of COPD related to tobacco smoking (COPD-S) and 190 with COPD related to biomass burning (COPD-BB). The COPD diagnosis was confirmed using pulmonary function tests, considering a ratio of forced expiratory volume in the first second/forced vital capacity (FEV_1_/FVC) < 70% as COPD according to the reference values for Mexicans obtained by Perez-Padilla *et al*.^21^. Individuals older than 40 years and with a tobacco index ≥5 packs/year were classified in the COPD-S group, and those older than 50 years with a biomass exposure index (exposure hours per day multiplied by years of exposure) higher than 30 h/year to biomass smoke were classified into the COPD-BB group. All included patients were stable, without supplementary oxygen at the recruitment moment, without a history of exacerbations at least in the previous three months, and without antibiotics and systemic corticosteroid treatment in the last 3 months. A depicted flowchart describing eligible and enrolled subjects is included in the supplementary information (Supplementary Fig. 1).

The COPD-S and COPD-BB groups were divided into two subgroups: frequent exacerbators (FE-S and FE-BB, respectively) (≥2 exacerbations per year) and non-exacerbators (NEX-S and NEX-BB, respectively) with emphysema or chronic bronchitis (<2 exacerbations per year) according to Spanish COPD Guidelines (GesEPOC)^22^. Additionally, exacerbations were diagnosed and classified according to Anthonisen Criteria^23^. In addition, GOLD stages I and II were grouped as G1 and stages III and IV as G2. Exacerbation history was obtained from medical records, and those patients who did not have information about exacerbations were excluded from this analysis.

The group of subjects without COPD (SWOC, n = 657) included smokers with no evidence of pulmonary disease and with normal spirometry parameters.

Finally, a group of contacts exposed to biomass burning, without a history of active or passive smoking, without evidence of pulmonary disease and normal spirometry values was also included (BBES, n = 183). Participants in this group are part of the national program for equality between women and men with the “Diagnóstico oportuno de EPOC/Respirar sin humo” campaign in women living in rural areas, primarily in the northern highlands of the state of Oaxaca and suburban areas of the Tlalpan mayoralty of Mexico City^24^.

All participants underwent a background questionnaire of inherited pathologies, whereby subjects who reported suffering some type of lung and/or chronic inflammatory disease were excluded, as well as subjects with non-Mexican ancestry (with no Mexican-born parents and grandparents).

#### Population comparison group

A group of 344 volunteers (healthy subjects, HS) was also included. These subjects had the following characteristics: clinically healthy (with neither chronic nor acute diseases self-reported), older than 20 years, both genders, non-smokers and no history of biomass burning smoke exposure, born as Mexican mestizo (MM, parents, and grandparents born in Mexico, no biological relation among themselves or with the patients or controls), and no history of family pulmonary diseases. The participants were recruited from annual campaigns of COPD early diagnosis performed on World COPD Day and World No-Smoking Day at the INER. However, these subjects did not undergo a pulmonary function test.

#### Ethics approval and informed consent

This study was approved by the Institutional Committees for Research, Ethics in Research, and Biosecurity of the Instituto Nacional de Enfermedades Respiratorias Ismael Cosío Villegas (INER) (approbation protocol codes: B10-12, B11-19). All participants were previously invited to participate in the study; they signed an informed consent document and were provided with a privacy statement describing the protection of personal data. Both documents were approved by the Research and Ethics in Research Committees of this institute.

All experiments were performed in accordance with the relevant guidelines and regulations. The STREGA (STrengthening the REporting of Genetic Association) guidelines were considered in the design of this genetic association study.

### DNA extraction

The DNA was extracted from peripheral blood cells via venipuncture from full 1,807 subjects included in this study using the commercial BDtract Genomic DNA isolation kit (Maxim Biotech, San Francisco, CA, USA). The DNA was then quantified by UV absorption spectrophotometry at the 260-nm wavelength using a NanoDrop system (Thermo Scientific, Wilmington, DE, USA). Contamination with organic compounds and proteins was determined by measuring the ratio absorbance at 280 nm and 260 nm. Samples were considered of good quality when the ratio was ~1.8.

### SNP selected

**Figure 1:**
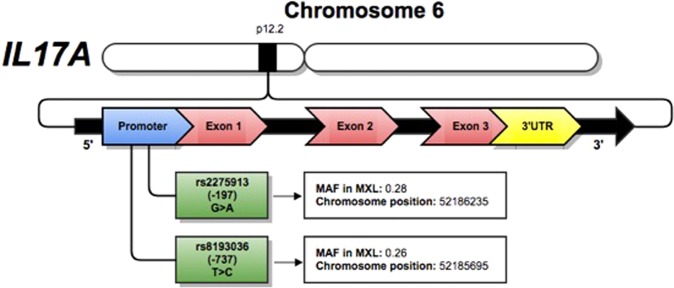
*IL17A* gene and characteristics of selected SNPs. MAF: minor allele frequency. MXL: Mexican from Los Angeles.

SNPs were selected based on a bibliographic search in PubMed (NCBI), identifying polymorphisms previously associated with other inflammatory and respiratory diseases, such as rheumatoid arthritis, systemic lupus erythematosus, psoriasis, tuberculosis, and asthma, and in previous reports in COPD. Additionally, we considered a minor allelic frequency (MAF) higher than 5% in the Mexican population in Los Angeles according to 1000 genomes^25^. Fig. 1 summarizes the principal characteristics of the evaluated SNPs.

### Genotyping of the SNPs

The allelic discrimination of SNPs was performed using commercial TaqMan probes (Applied Biosystems, California, USA) at a concentration of 20X in total subjects included. SNPs evaluated were rs2275913 and rs8193036 (*IL17A*) using qPCR in a 7300 Real-Time PCR System (Applied Biosystems, CA, USA), and the analysis was performed by SDS (sequence detection software) version 1.4 software (Applied Biosystems, CA, USA). In addition, three controls without template (contamination controls) were included for each genotyping plate, and 1% of the samples included in the study were genotyped in duplicate as controls for allele assignment.

### Measurement of serum levels of IL-17A

After performing the association analyses, we selected 39 serum samples from the COPD-S, 38 SWOC, 37 COPD-BE and 38 BBES carrying alleles of rs8193036 associated with the risk of COPD. Sample selection was performed to identify representability for each genotype from the associated SNP. In the COPD-S group, we selected 15 with TT, 10 with TC and 14 with CC genotypes. In the SWOC group, we selected 16 with TT, 8 with TC and 14 with CC genotypes. For the biomass burning-exposed groups, in the COPD-BB group, we selected 18 with TT, 15 with TC and 4 with CC genotypes. In the BBES group, we selected 20 with TT, 12 with TC and 6 with CC genotypes. The differences in the number of patients according to genotypes are due to the availability of serum samples and the genotype frequencies. These serum levels were measured by ELISA, and the minimum detectable concentration of IL-17A was 0.7 pg/mL (Cat. No. 433915, Biolegend San Diego, CA). A total of 50 µL of plasma for each sample was centrifuged and prepared for analysis. The manufacturer’s protocol was followed.

### Statistical analysis

The differences between groups under study were evaluated by determining and comparing the allele, and genotype frequencies. Statistical significance was assessed using SPSS v20.0 (SPSS Inc., Chicago, IL, USA) and Epi Info 7.1.4.0 (Centers for Disease Control and Prevention, Atlanta, GA, USA) statistical software. To determine the possible associations between the analyzed polymorphisms, the allele and genotype frequencies were analyzed between groups using the χ^2^ test. The results were considered to be significant when the *p*-value was <0.05; similarly, the odds ratios (ORs) with 95% confidence intervals (CI) were estimated to determine the strength of the association.

Comparisons were made between cases (COPD-S and COPD-BB) and controls (SWOC and BBES) groups. In addition, another comparison was made between cases and the healthy subjects’ group.

A logistic regression analysis was performed to adjust for potential confounding variables (sex, age, tobacco index, biomass-exposure index) using Plink v. 1.07^26^.

## Results

### Demographic variables

#### COPD related to tobacco smoking and biomass burning

The demographic and pulmonary function data of COPD-S, SWOC, COPD-BB, BBES, and HS are presented in Table 1. Age and sex were significantly different between COPD-S and COPD-BB patients *vs*. SWOC and BBES groups, respectively (COPD groups are older than the controls, and the COPD-S group is predominantly men, p < 0.01). Based on these findings, we decided to perform a logistic regression analysis by co-variables that resulted in significant differences to eliminate potential confounding variables. The results of this analysis are described below. Interestingly, the biomass exposure index (BEI) was lower in the COPD-BB group than in the BBES group. However, there were no significant differences in this parameter. COPD-S patients had more years smoking than smokers without COPD, as well as packs per year history (p < 0.01). However, the onset of smoking did not show differences between these groups (p = 0.98). As expected, in pulmonary function variables (by spirometry test), there was a statistically significant result (p < 0.01) between COPD-S and SWOC groups, as well as COPD-BB *vs*. BBES (p < 0.01), in each variable.

**Table 1:** Demographic variables and pulmonary function data from all five groups.

Variables	COPD-S (n = 439)	SWOC (n = 657)	*p-*value	COPD-BB (n = 190)	BBES (n = 183)	*p-*value	HS (n = 341)
Age, years	67 (40–93)	53 (38–90)	<0.01	73 (51–97	63 (41–85)	<0.01	55 (21–80)
Female (%)	26.38	51.60	<0.01	89.47	98.92	<0.01	76.19
**Biomass Exposure status**							
Hours per day exposed				7 (1–24)	5 (1–12)	<0.01	
Years exposed				40 (3–82)	46 (5–80)	0.12	
Biomass Exposure Index				255 (36–960)	272 (12–864)	0.23	
**Smoking status**							
Years of smoking	43 (10–73)	31 (10–60)	<0.01				
Cigarettes per day	20 (3–80)	20 (8–80)	0.03				
Packs-year history	40 (5–200)	27 (5–168)	<0.01				
Onset of smoking	16 (6–65)	17 (5–49)	0.98				
**Pulmonary function**							
FVC (%) post	83 (18–151)	95 (29–156)	<0.01	86 (57–131)	101 (62–193)	<0.01	
FEV1 (%) post	54 (15–119)	98 (37–160)	<0.01	61 (30–114)	104 (60–187)	<0.01	
FEV1/FVC (%) post	54 (19–69)	82 (70–113)	<0.01	55 (31–69)	80 (71–121)	<0.01	

COPD-S: Patients with COPD related to tobacco smoking, SWOC: Smokers without COPD, COPD-BB: Patients with COPD related to biomass burning, BBES: Biomass burning-exposed subjects, HS: Healthy subjects.

All values are measures of pulmonary function post-bronchodilator use. A p-value < 0.05 was significant. We used the median test to make comparisons between groups and data are shown as the minimum and maximum values.

#### COPD related to tobacco smoking and biomass burning and exacerbation frequency

Additionally, within the COPD-S and COPD-BB groups, we compared frequent exacerbators (FE-S and FE-BB, respectively) against non-exacerbators (NEX-S and NEX-BB, respectively). The results are presented in Table 2. Age and sex did not show significant results between groups (p = 0.85 and p = 0.86, respectively). Regarding BEI, there were no significant differences between FE-BB *vs*. NEX-BB groups. Referring to smoking status, years of smoking, cigarettes per day and packs per year history were similar in both groups (FE-S *vs*. NEX-S, p > 0.05). Interestingly, the onset of smoking showed a statistically significant difference (p < 0.01), with frequent exacerbators who started smoking at earlier ages. GOLD l + ll (G1) were more prevalent in NEX-S than FE-S, while GOLD lll and lV (G2) were more frequent in the FE-S group (p < 0.01). However, there were no important differences when comparing G1 and G2 from the FE-BB and NEX-BB groups. Also, the FE-S group had a worse pulmonary function than NEX-S, reflected in FVC% (p = 0.04), FEV1% (p < 0.01) and FEV1/FVC (p = 0.01).

**Table 2:** Demographic variables among COPD patients FE-S, NEX-S, FE-BB and NEX-BB.

Variables	FE-S (n = 71)	NEX-S (n = 149)	*p-*value	FE-BB (n = 50)	NEX-BB (n = 85)	*p-*value
Age, years	68 (51–93)	67 (42–93)	0.85	70 (58–90)	76 (51–97)	0.10
Female (%)	21.13	22.82	0.86	88.24	90.59	0.66
**Biomass Exposure status**						
Hours per day exposed				6 (2–18)	7 (1–24)	0.54
Years exposed				40 (8–75)	40 (7–80)	0.89
Biomass Exposure Index				225 (60–800)	205 (48–960)	0.98
**Smoking status**						
Years of smoking	45 (15–65)	42 (10–64)	0.36			
Cigarettes per day	20 (4–80)	20 (5–80)	0.99			
Packs-year history	40 (8–200)	40 (5–200)	0.19			
Onset of smoking	15 (6–25)	17 (7–60)	0.01			
**GOLD**						
G1 (I - II) [%]	31.15	65.71	<0.01	70.59	68.67	0.88
G2 (III - IV) [%]	68.85	34.29		29.41	31.33	
**Pulmonary function**						
FVC (%) post	80 (42–139)	86 (18–155)	0.04	95 (57–178)	87 (61–131)	0.61
FEV1 (%) post	42 (18–108)	57 (18–123)	<0.01	65 (34–130)	65 (30–114)	0.83
FEV1/FVC (%) post	44 (24–69)	54 (19–69)	0.01	56 (31–69)	58 (36–69)	0.90

FE-S = Patients with COPD related to tobacco smoking frequent exacerbators, NEX-S = Patients with COPD related to tobacco smoking non-exacerbators, FE-BB = Patients with COPD related to biomass burning frequent exacerbators, NEX-BB = Patients with COPD related to biomass burning non-exacerbators. G1 = GOLD I and GOLD II stages, G2 = GOLD III and GOLD IV stages. All values are measures of pulmonary function post-bronchodilator use. A p-value < 0.05 was significant. We used median and Mann-Whitney U tests to make comparisons between groups. Data are shown as the minimum and maximum values.

#### Analysis of the allele and genotype association

Two SNPs (rs2275913 and rs8193036) were evaluated in the *IL17A* gene. The results of the allele and genotype frequencies are presented in Table 3.

**Table 3:** Analysis of the association of alleles and genotypes in co-dominant, dominant and recessive models.

SNP/Model	COPD-S		SWOC		COPD-BB		BBES		HS		*p-value*	OR	95% CI
	n = 436	F (%)	n = 657	F (%)	n = 190	F (%)	n = 183	F (%)	n = 344	F (%)			
**rs2275913**													
Co-dominant													
GG	275	63.07	423	64.38	133	70	134	73.22	243	71.3	1		
GA	141	32.34	210	31.96	56	29.47	46	25.14	93	27.3	<0.01†	1.33	0.97–1.83
AA	20	4.59	24	3.65	1	0.53	3	1.64	5	1.47		3.53	1.30–9.56
Alleles													
G	691	79.24	1056	80.37	322	84.74	314	85.79	579	84.90	<0.01†	1.47	1.12–1.92
A	181	20.76	258	19.63	58	15.26	52	14.21	103	15.10			
Dominant													
GG	275	63.07	423	64.38	133	70	134	73.22	243	71.26	0.02†	1.45	1.07–1.96
GA + AA	161	36.93	234	35.62	57	30	49	26.78	98	28.74			
Recessive													
GG + GA	416	95.41	633	96.35	189	99.47	180	98.36	336	98.53	0.01†	3.23	1.19–8.69
AA	20	4.59	24	3.65	1	0.53	3	1.64	5	1.47			
**rs8193036**													
Co-dominant													
TT	259	59.40	420	63.93	118	62.11	115	62.16	199	57.9	1		
TC	151	34.63	223	33.94	67	35.26	63	34.05	125	36.3	0.02^§^	1.09	0.84–1.42
CC	26	5.96	14	2.13	5	2.63	7	3.78	20	5.81		3.01	1.54–5.87
Alleles													
T	669	76.72	1063	80.90	303	79.74	293	79.19	523	76	0.02^§^	1.28	1.04–1.59
C	203	23.28	251	19.10	77	20.26	77	20.81	165	24			
Dominant													
TT	259	59.40	420	63.93	118	62.11	115	62.16	199	57.85	NS		
TC + CC	177	40.60	237	36.07	72	37.89	70	37.84	145	42.15			
Recessive													
TT + TC	410	94.04	643	97.87	185	97.37	178	96.22	324	94.19	<0.01^§^	2.91	1.50–5.64
CC	26	5.96	14	2.13	5	2.63	7	3.78	20	5.81			

^†^When comparing COPD-S versus HS. ^§^When comparing COPD-S versus SWOC. COPD-S: Patients with COPD related to tobacco smoking, SWOC: Smokers without COPD, COPD-BB: Patients with COPD related to biomass burning, BBES: Biomass burning-exposed subjects, HS: Healthy subjects, F: Frequency, NS: Nonsignificant.

#### COPD patients and healthy subjects’ comparison

In the co-dominant model, a statistically significant difference was found for the AA genotype of rs2275913 when comparing COPD-S *vs*. HS (p < 0.01; OR = 3.53). Supporting this finding, the A allele for the same SNP was also associated with the presence of COPD related to tobacco smoking (p < 0.01; OR = 1.47). Additionally, dominant and recessive models were associated with COPD in the same comparison (p = 0.02; OR = 1.45 and p = 0.01, OR = 3.23, respectively). This comparison is shown in Table 3.

Comparisons between COPD-BB, BBES, and HS did not show statistically significant differences.

#### Case-control comparisons

Regarding rs8193036, genotype CC was associated with COPD when comparing COPD-S *vs*. SWOC (p = 0.02; OR = 3.01). The C allele also showed a statistically significant difference in the same comparison (p = 0.02; OR = 1.28). The recessive model was also evaluated and was found to be associated with susceptibility to COPD (p < 0.01; OR = 2.91), as shown in Table 3.

Logistic regression was performed to adjust for co-variables. For rs8193036/C, we found statistically significant differences comparing COPD-S *vs*. SWOC adjusting by sex (p = 9.36E-06, OR = 2.12, CI 95% 1.519–2.947) and age (p = 1.89E-39, OR = 1.15, CI 95% 1.122–1.168). Additionally, for rs2275913/A, adjusting by sex and age, we found significant differences for the same comparison (p = 9.49E-06, OR = 2.11, CI 95% 1.518–2.95; p = 8.91E-40, OR = 1.15, CI 95% 1.123–1.17, respectively). The results are presented in Supplementary Table 1.

Analysis by genotype showed that rs8193036/CC was associated with a risk of COPD related to tobacco smoking in an additive model (p = 0.02, OR = 1.68, CI 95% 1.10–2.57) and adjusting for age (p = 1.94E-58, OR = 1.16, CI 95% 1.14–1.18). However, in the dominant model, we found significant differences for reduced risk (p = 0.02, OR = 0.57, CI 95% 0.34–0.93).

For rs2274913/AA, we only found a significant difference for risk to COPD adjusting by age (p = 7.00E-59, OR = 1.16, CI 95% 1.14–1.18). The results are presented in Supplementary Table 2.

Contrary to the unadjusted analysis, both SNPs were found to be associated with an increased risk of COPD among those exposed to biomass-burning smoke. After performing the logistic regression analysis (Supplementary Table 3), we found significant differences in rs8193036/C, comparing COPD-BB *vs*. BBES by sex (p = 3.68E-04, OR = 18.94, CI 95% 3.754–95.54) and age (p = 4.52E-17, OR = 1.11, CI 95% 1.08–1.13). However, for rs2275913/A after adjusting for sex (p = 5.06E-04, OR = 17.78, CI 95% 3.51–90.02) and age (p = 3.15E-17, OR = 1.11, CI 95% 1.084–1.138).

Interestingly, we found significant differences in the risk of COPD related to biomass burning after adjusting for age for rs8193036 and rs2275913 (p = 7.46E-17, OR = 1.10, CI 95% 1.10–1.13; and p = 5.55E-17, OR = 1.10, CI 95% 1.10–1.13, respectively). The results are presented in Supplementary Table 4.

#### Allele and genotype association with exacerbations in COPD related to tobacco smoking- and biomass burning-exposed subjects

A comparison between FE-S *vs*. NEX-S and FE-BB *vs*. NEX-BB was performed. We evaluated the same two SNPs for the *IL17A* gene. Allele and genotype frequencies did not show statistically significant differences between groups, and neither did co-dominant, dominant and recessive models. The results are shown in Table 4.

**Table 4:** Analysis of the association of alleles and genotypes in co-dominant, dominant and recessive models between frequent exacerbators and non-exacerbators.

Gene/SNP	Model	Genotype/Allele	FE-S		NEX-S		p-value	OR	95% CI	FE-BB		NEX-BB		p-value	OR	95% CI
			n = 71	F (%)	n = 149	F (%)				n = 50	F (%)	n = 85	F (%)			
***IL17A***																
rs2275913	Co-dominant	GG	40	56.34	97	65.10	1			37	74	56	65.88			
		GA	28	39.44	47	31.54	0.24	1.44	0.79–2.62	13	26	29	34.12	NA		
		AA	3	4.23	5	3.36		1.45	0.33–6.37	0	0	0	0			
	Alleles	G	108	72.97	241	80.87	0.07	1.56	0.98–2.48	87	87	141	82.94	0.49	0.72	0.36–1.45
		A	40	27.03	57	19.13				13	13	29	17.06			
	Dominant	GG	40	56.34	97	65.10	0.24	1.44	0.81–2.57	37	74	56	65.88	0.34	0.67	0.31–1.47
		GA + AA	31	43.66	52	34.90				13	26	29	34.12			
	Recessive	GA + GG	68	95.77	144	96.64	0.71	1.27	0.29–5.47	50	100	85	100	NA		
		AA	3	4.23	5	3.36				0	0	0	0			
										n = 51	F (%)	n = 84	F (%)			
rs8193036	Co-dominant	TT	44	61.97	95	63.76	1			25	49.02	55	65.48			
		TC	22	30.99	45	30.20	0.75	1.05	0.56–1.96	26	50.98	27	32.14	NA		
		CC	5	7.04	9	6.04		1.19	0.37–3.78	0	0	2	2.38			
	Alleles	T	110	75.86	325	78.86	0.47	1.18	0.74–1.90	76	74.51	137	81.55	0.22	1.51	0.83–2.73
		C	35	24.14	73	21.14				26	25.49	31	18.45			
	Dominant	TT	44	61.97	95	63.76	0.88	1.07	0.60–1.93	25	49.02	55	65.48	0.07	1.9	0.96–4.01
		TC + CC	27	38.03	54	36.24				26	50.98	29	34.52			
	Recessive	TT + TC	66	92.96	140	93.96	0.77	1.17	0.38–3.65	51	100	57	67.86	NA		
		CC	5	7.04	9	6.04				0	0	27	32.14			

COPD-S (FE) = Patients with COPD related to smoking frequent exacerbators, COPD-S (NEX) = Patients with COPD related to tobacco smoking non-exacerbators; NA: Not applicable due to null values (0).

However, after adjusting for the onset of smoking (Supplementary Table 1), we found a significant association with a reduced risk of rs8193036/C comparing FE-S *vs*. NEX-S (p < 0.03, OR = 0.93, CI 95% 0.87–0.99) and rs2275913 (p < 0.03, OR = 0.92, CI 95% 0.87–0.99). Additionally, adjusting for age, we found a significant association with a reduced risk of the rs8193036/CC genotype (p < 0.03, OR = 0.93, CI 95% 0.88–0.99) and rs2275913/AA (p < 0.03, OR = 0.93, CI 95% 0.88–0.99). The results are shown in Supplementary Table 2. In addition, in FE-BB *vs*. NEX-BB, adjusting for age, we found a reduced risk for frequent exacerbations with rs8193036/C (p = 0.04, OR = 0.95, CI 95% 0.91–0.99) and rs2275913/A (p = 0.03, OR = 0.96, CI 95% 0.91–0.99). The results are shown in Supplementary Table 3. We did not find a significant association after performing a regression analysis for risk to exacerbations with genotypes for either SNP comparing FE-BB *vs*. NEX-BB.

### IL-17A serum levels and genotype

In general, serum IL-17A concentration was considerably higher in those patients who were exposed to tobacco smoking than those exposed to biomass burning. Serum levels in the COPD-S, SWOC, COPD-BB and BBEES groups were 4.4 ± 1.6 pg/mL, 4.1 ± 0.9 pg/mL, 2.3 ± 1.2 pg/mL and 2.5 ± 1.1 pg/mL, respectively.

**Figure 2:**
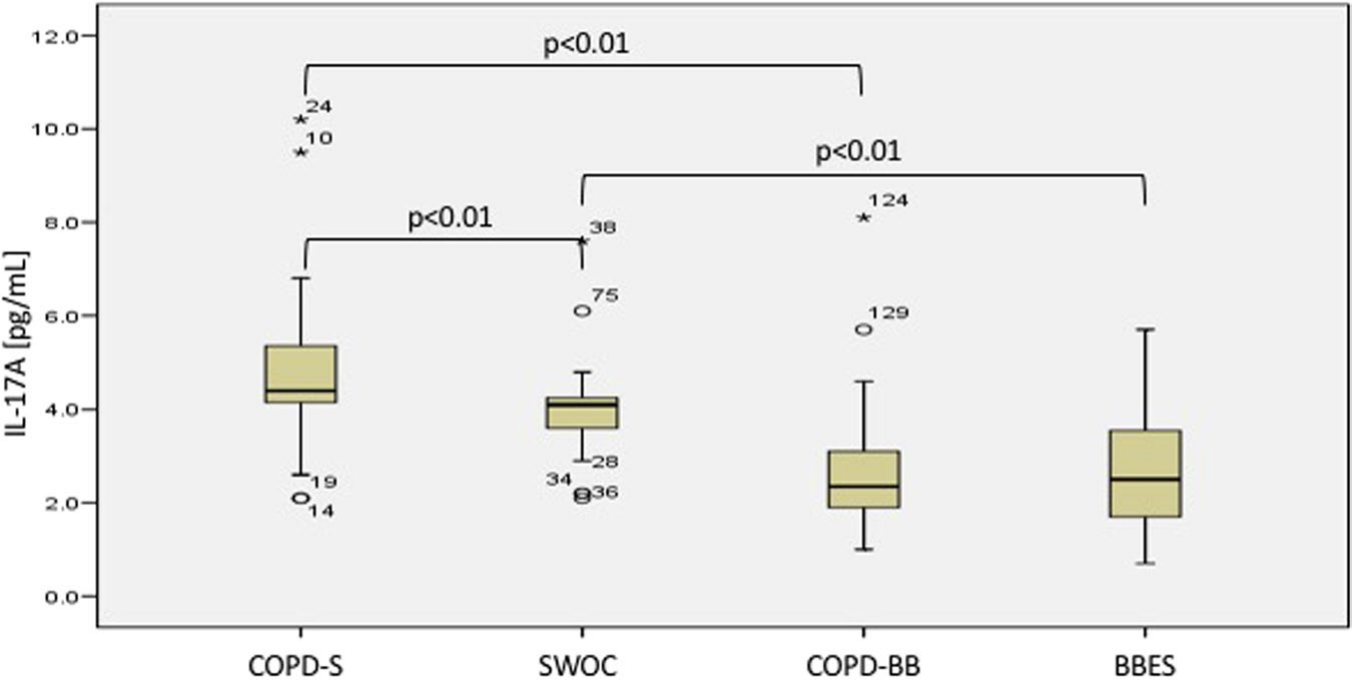
IL-17A serum levels in COPD groups and controls. COPD-S: COPD related to tobacco smoking; SWOC: smokers without COPD; COPD-BB: COPD related to biomass burning exposure; BBES: biomass burning-exposed subjects.

We found significant differences when comparing serum levels between COPD-S (4.4 ± 1.6 pg/mL) *vs*. SWOC (4.1 ± 0.9 pg/mL; p < 0.01), COPD-S (4.4 ± 1.6 pg/mL) *vs*. COPD-BB (2.3 ± 1.2 pg/mL; p < 0.01), and SWOC (4.1 ± 0.9 pg/mL) *vs*. BBES (2.5 ± 1.1 pg/mL; p < 0.01). We did not find significant differences between groups exposed to biomass burning. Differences are presented in Fig. 2.

**Figure 3:**
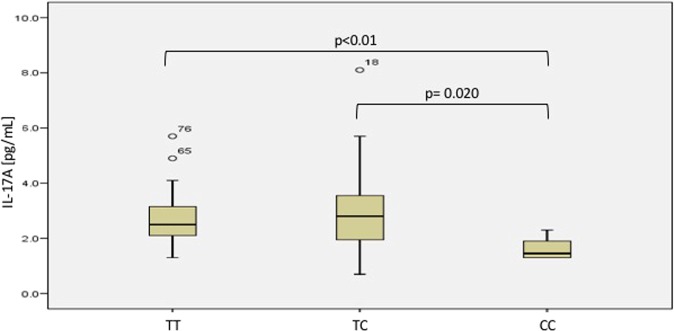
IL-17A serum levels between genotypes of rs8193036 in the COPD-BB group; COPD-BB: COPD related to biomass burning exposure.

In addition, by comparing genotypes, we found an association in the COPD-BB group when comparing TT *vs*. CC and TC *vs*. CC (p < 0.01 and p = 0.02, respectively), with lower levels being observed for the CC genotype. The results are presented in Fig. 3. We did not find significant differences by genotype in the COPD-S group. IL-17A serum levels are shown in Supplementary Fig. 2.

## Discussion

COPD is a complex and multifactorial disease in which one of its primordial mechanisms in pulmonary lesions is an inflammatory component, as well as a widely described exposure to environmental risk factors and genetic predisposition.

Regarding demographic variables, our results have a classical behavior, determining that COPD related to smoking is more prevalent in male and older people and that COPD related to biomass burning is more prevalent in older women. Additionally, as expected, COPD cases had worse pulmonary function than subjects with environmental risk factor exposure without the illness. Also, when we compared frequent exacerbators *vs*. non-exacerbators, we found that patients who have recurrent exacerbations have a worse pulmonary function; hence, they are in more advanced stages, resulting in greater progression of the disease.

It is known that exacerbations result in a negative impact on the quality of life of patients with COPD and can cause acceleration of disease progression and frequent hospital admissions. Tanabe *et al*.^27^ demonstrated this by computed tomography (CT) taken every year for two years. These researchers found that those subjects who had one or more exacerbations increased the percentage of low-attenuation areas in CT, suggesting emphysema progression.

Additionally, exacerbations are the most common cause of death of patients with COPD, reaching up >6% 90 days after the episode^28–31^. It has been previously reported in such large prospective cohorts as ECLIPSE and SPIROMICS that exacerbations are associated with worse pulmonary function and gravity of the disease; specifically, those patients who are in GOLD III and IV stages have more exacerbations than those who are in GOLD I and II stages^32–34^. It is important to note that exacerbations according to the Lung Health Study contribute to the FEV1 decline of 7 mL per year^35^. Previously, several studies have reported clinical predictors for exacerbation frequency, such as age, disease severity, use of inhaled corticosteroids, and poor pulmonary function^36,37^. However, we describe for the first time that COPD patients who start smoking at an early age have a higher risk for frequent exacerbations.

These findings agree with our results and reflect the difficulty in classifying these patients and how complex this pathology is. However, it is true that exacerbations depend on many factors, such as adequate adherence to base treatment and vaccination, and are more frequent in certain seasons of the year. Additionally, Mei Lan *et al*.^32^ report that COPD is highly variable and that in a follow-up of three years, only a low percentage remains as FE, and those who are classified as NEX can also change to FE^37^.

Regarding COPD related to biomass burning, there were no significant differences in the biomass exposure index between COPD-BB and BBES, and it was even slightly higher in the BBES group. This finding can be explained because despite biomass exposure is associated with COPD risk, there are other factors that could play an important role in COPD development, such as genetic susceptibility, wide but not completely described until now.

Several studies have been conducted in proinflammatory genes, and an association with the presence of COPD has been found, but investigations of the Th17 profile are notably scarce. It has been previously reported that in the Tatar population, the *IL17A* rs1974226 had a significant association with COPD, as well as smoking status (major packs per year)^38^. In comparison with our results, rs8193036 and rs2275913 are also associated with COPD. In contrast with our findings, Dai and colleagues^39^ described that in a Chinese population, individuals carrying the rs2275913 A allele had a reduced risk of presenting COPD related to tobacco smoking. However, this may be because the sample size was considerably higher in our study or due to the differences in genetic contribution.

Little is known about the genetic background of COPD related to biomass-burning smoke exposure. There are few studies that describe clinical outcomes and genetic components. Recently, Reséndiz-Hernández *et al*.^40^ described for the first time that some polymorphisms and haplotypes in the *TNF* promoter confer risk for COPD related to biomass burning exposure. In our study, we did not find any association with *IL17A* polymorphisms for unadjusted analysis. Nevertheless, logistic regression analysis revealed an association between both SNPs and risk for COPD related to biomass burning exposure. Markedly differences exist in COPD related to biomass burning and tobacco smoking. For example, we already know that biomass-exposure COPD patients have a slower rate of FEV1 decline and show a more homogeneous rate of decline in comparison with COPD related to smoking^41^.

These results indicate that these genetic variants in Th17 profile genes may play a key role in pulmonary disease pathogenesis, including COPD. Additionally, other studies have revealed an increase in Th17-related cytokine expression in biological samples, such as blood and sputum, in stable COPD and during exacerbations in comparison with smokers with normal pulmonary function^42–44^. Indeed, some studies have shown an increased expression of ROR-γT in lung tissue, which is necessary for Th17 polarization. On the other hand, a lower expression of cytokines related to regulatory T cells (Tregs) has been observed in patients with COPD and exacerbations. This imbalance, in theory, promotes an altered response in the lung parenchyma causing damage.

Interestingly, it has been demonstrated that rs2275913 (G > A) is a functional polymorphism that allows stronger binding of NFAT (nuclear factor of activated T-cells), a transcriptional factor, to the *IL17A* promoter, leading to higher transcription and synthesis of the IL-17A protein^45^. IL-17A promotes chemotaxis to the inflammation zone (especially attracting neutrophils). This effect leads to lung tissue damage and the progression of the disease. Thus, it is feasible to believe that the higher the concentration is, the greater the neutrophilia is. Supporting this idea, Roos and coworkers^46^ demonstrated in their study that during Non-typeable *Haemophilus influenzae* infection, IL-17A concentration in sputum was higher in COPD patients during the exacerbation than before and after the exacerbation. Additionally, in the same study with a *knockout* murine model, they suppressed *Il17a* and observed that neutrophilia was attenuated in comparison with wild-type mice, suggesting an important role of this cytokine in neutrophilic inflammation^46^. This finding suggests that IL-17A could be (in the future) a novel therapeutic target to attenuate neutrophilia present in airways in acute exacerbations^47^. However, more investigations are required.

Few studies have been carried out to find genetic susceptibility to exacerbations in patients with COPD. The results have been contradictory, but it is necessary to continue generating knowledge about the exacerbator phenotype. In a Japanese population, it has been described that group component (*GC*) (a protein that binds to vitamin D and affects its concentration) polymorphisms are associated with frequent exacerbations, as well as COPD presence and a tendency for fast pulmonary function decline^48^. Additionally, other genes have been evaluated in different populations, such as *SFTPB* (Surfactant B protein), *MBL* (mannose-binding lectin) *TNF* and *SIGLEC9* (Sialic acid-binding Ig-like lectin 9), which were associated with frequent exacerbations^49–52^.

Our results of the unadjusted analysis show a tendency, whereby we believe that if we increase the sample size, we could find an association with rs2275913; therefore, we do not rule out that *IL17A* polymorphisms are associated with frequent exacerbations. This finding tells us about the complex interaction between several factors such that exacerbations can occur. Conversely, after logistic regression analysis, the results showed that both SNPs are associated with a reduced risk for COPD exacerbations by alleles (rs2275913 and rs8193036) and genotypes (rs2275913), which has not been previously reported.

Regarding serum levels, in our study, we observed a greater concentration of IL-17A in COPD-S than SWOC, and we found significant differences in those smokers (smokers without COPD and COPD-S) *vs*. biomass burning-exposed subjects (with and without COPD). Several studies have demonstrated similar findings to ours. For example, Jiang *et al*.^53^ showed in their study that serum levels of IL-17 were considerably higher in patients with worse severity of disease (stages GOLD III and IV), relating to the progression of the disease. Additionally, Zou *et al*.^54^ found that serum IL-1β and IL-17 levels were significantly higher in patients with COPD than in the healthy control group. Little is known about the immunopathogenesis of COPD related to biomass-burning smoke exposure. In a study conducted by Golpe and colleagues^55^, described some differences between COPD patients depending on the exposure agent. Higher serum levels of IL-8, IL-6 and IL-5 were found in patients with COPD related to tobacco smoking than COPD related to biomass burning exposure. Other molecules implicated in the innate immune response have been deeply reviewed^56^. Also, clinical and inflammatory markers in patients with COPD related to tobacco smoking *vs*. biomass burning exposure COPD patients are different regarding the source of exposure. For example, COPD related to biomass burning had significantly higher IgE serum levels and lower C reactive protein (CRP) serum levels^57^. These results suggest an important role for the Th2 profile in the pathogenesis of COPD related to biomass-burning smoke exposure.

On the other hand, there are no previous reports about a clear relationship between the Th17 profile and COPD related to biomass burning. In our study, we found an allele and genotype association to consider rs2275913 and rs8193036 as a risk factor for developing COPD in subjects exposed to biomass burning, and we found lower levels of IL-17A than smokers. However, compared with genotypes, we found higher levels of TT and TC genotypes than CC (homozygous to minor allele). Decreased levels of IL-17A in COPD-BB and BBES could reflect a differential systemic inflammatory pattern triggered by biomass smoke.

For everything mentioned above, there is enough evidence to believe that the Th17 profile, especially IL-17A, could play a crucial role in pulmonary immunity, participating in a large number of chronic respiratory diseases, such as COPD, promoting and perpetuating lung inflammation and generating lung injury. Additionally, IL-17A could participate in response to microorganisms that could trigger exacerbations of the disease. More studies are required to elucidate the immunological, genetic and pathologic mechanisms governing COPD and exacerbations and to understand them better.

There were some limitations to the current study. The sample size was reduced when the exacerbations frequency analysis was carried out due to missed data on clinical records. The serum determinations were done at a single cross-sectional time point. Nevertheless, the protein levels were stratified by the genotype of the associated SNPs to explore the relationship between protein levels and genotypes. Although the inclusion of patients under treatment represented another limitation, this was done to avoid limiting the analysis to patients with less severe forms of the disease.

Finally, this study has important strengths. We include large sample size and two comparison groups (COPD related to smoking and biomass-burning smoke exposure). Also, Th17 profile (genetic and protein levels) has been evaluated in COPD related to biomass-burning smoke exposure for the first time. Interestingly, rs2275913 and rs8193036 maintained their association before and after adjusted analysis.

## Conclusions

rs2275913 and rs8193036 in *IL17A* are associated with the risk of COPD related to tobacco smoking, especially when they are in the homozygous state. After logistic regression analysis, associations between SNPs and risk for COPD are maintained, and new associations with risk for COPD related to biomass burning exposure were found, as well as a reduced risk for exacerbations, were observed. Additionally, serum levels of IL-17A were higher in smokers with and without COPD than those exposed to biomass burning with and without the disease. Further investigations are required to elucidate immunopathogenic mechanisms in COPD and COPD exacerbations that have remained unclear; these studies may investigate treatment alternatives to improve the prognosis of patients.

## Supplementary information

Supplementary information.

## Data Availability

Data will be made available on reasonable request.
