# Supplementary material for: Genetic variants in IL17A and serum levels of IL-17A are associated with COPD related to tobacco smoking and biomass burning
Source: Sci Rep. 2020 Jan 21;10:784. doi: 10.1038/s41598-020-57606-6 (PMC6972744; doi:10.1038/s41598-020-57606-6)
Supplement: Supplementary file 1 — Supplementary information. [file 41598_2020_57606_MOESM1_ESM.pdf]

Title

Genetic variants in *IL17A* and serum levels of IL-17A are associated with COPD related to tobacco smoking and biomass burning

Authors

Marco A. Ponce-Gallegos, Gloria Pérez-Rubio, Enrique Ambrocio-Ortiz, Neftali Partida-Zavala, Rafael Hernández-Zenteno, Fernando Flores-Trujillo, Leonor García-Gómez, Andrea Hernández-Pérez, Alejandra Ramírez-Venegas, Ramcés Falfán-Valencia

Supplementary tables

Supplementary Table 1. Logistic regression analysis by co-variables and alleles in COPD-S group.

COPD-S comparison (Alleles)											
CHR	SNP	BP	A1	TEST	NMISS	OR	SE	L95	U95	STAT	p-value
6	rs8193036	52185695	2	ADD	1050	1.158	0.144	0.8736	1.536	1.021	0.3071
6	rs8193036	52185695	2	SEX	1050	2.115	0.1691	1.519	2.947	4.431	**9.36E-06**
6	rs8193036	52185695	2	COV1	1050	1.145	0.01027	1.122	1.168	13.14	**1.89E-39**
6	rs8193036	52185695	2	COV2	1050	1.008	0.01545	0.9779	1.039	0.5148	0.6067
6	rs8193036	52185695	2	COV3	1050	0.9977	0.02342	0.953	1.045	-0.0974	0.9224
6	rs8193036	52185695	2	COV4	1050	1.012	0.01236	0.9876	1.037	0.9543	0.3399
6	rs2275913	52186235	1	ADD	1050	1.196	0.1429	0.9034	1.582	1.25	0.2114
6	rs2275913	52186235	1	SEX	1050	2.115	0.1691	1.518	2.946	4.429	**9.49E-06**
6	rs2275913	52186235	1	COV1	1050	1.145	0.01028	1.123	1.169	13.2	**8.91E-40**
6	rs2275913	52186235	1	COV2	1050	1.008	0.0155	0.9777	1.039	0.5016	0.616
6	rs2275913	52186235	1	COV3	1050	0.9976	0.02343	0.9529	1.045	-0.101	0.9195
6	rs2275913	52186235	1	COV4	1050	1.012	0.01238	0.9876	1.037	0.9534	0.3404

cov1 Age

cov2 Years of smoking

cov3 Cigarettes per day

cov4 Tobacco index

Exacerbations among COPD-S (Alleles)											
CHR	SNP	BP	A1	TEST	NMISS	OR	SE	L95	U95	STAT	p-value
6	rs8193036	52185695	2	COV1	211	0.9294	0.03288	0.8714	0.9913	-2.227	**0.02597**
6	rs2275913	52186235	1	COV1	211	0.9286	0.03315	0.8701	0.9909	-2.236	**0.02537**

cov1 Onset of smoking

Abbreviations and keys: COPD-S: Patients with COPD related to tobacco smoking, CHR: Chromosome, SNP: Single Nucleotide Polymorphism, BP: Base pair location, A1: minor allele for each SNP, NMISS: No-missing data, OR: Odds ratio, SE: Standard error, L95: Lower interval confidence, U95: Upper interval confidence, ADD: Additive effect model, COV1-4: Covariables.

Supplementary Table 2. Logistic regression analysis by co-variables and genotypes in the COPD-S group.

COPD-S comparison (Genotypes)											
CHR	SNP	BP	A1	TEST	NMISS	OR	SE	L95	U95	STAT	p-value
6	rs8193036	52185695	2	ADD	1093	1.676	0.2185	1.092	2.572	2.363	**0.01814**
6	rs8193036	52185695	2	DOMDEV	1093	0.5659	0.2548	0.3435	0.9325	-2.234	**0.02546**
6	rs8193036	52185695	2	COV1	1093	1.161	0.009268	1.14	1.182	16.12	**1.94E-58**
6	rs8193036	52185695	2	GENO_2DF	1093	NA	NA	NA	NA	6	**0.04979**
6	rs2275913	52186235	1	ADD	1093	1.21	0.1974	0.822	1.782	0.9669	0.3336
6	rs2275913	52186235	1	DOMDEV	1093	0.8886	0.2411	0.554	1.425	-0.4901	0.6241
6	rs2275913	52186235	1	COV1	1093	1.161	0.00922	1.14	1.182	16.18	**7.00E-59**
6	rs2275913	52186235	1	GENO_2DF	1093	NA	NA	NA	NA	1.025	0.599

Exacerbations among COPD-S (Genotypes)											
CHR	SNP	BP	A1	TEST	NMISS	OR	SE	L95	U95	STAT	p-value

6	rs8193036	52185695	2	ADD	211	1.161	0.3087	0.6339	2.126	0.4833	0.6289
6	rs8193036	52185695	2	DOMDEV	211	0.9257	0.4077	0.4164	2.058	- 0.1894	0.8498
6	rs8193036	52185695	2	COV1	211	0.9334	0.03132	0.8778	0.9925	-2.201	**0.02776**
6	rs8193036	52185695	2	GENO_2DF	211	NA	NA	NA	NA	0.2532	0.8811
6	rs2275913	52186235	1	ADD	211	1.1	0.38	0.5225	2.317	0.2516	0.8013
6	rs2275913	52186235	1	DOMDEV	211	1.326	0.4532	0.5452	3.222	0.6218	0.5341
6	rs2275913	52186235	1	COV1	211	0.9314	0.0318	0.8751	0.9913	-2.235	**0.0254**
6	rs2275913	52186235	1	GENO_2DF	211	NA	NA	NA	NA	1.468	0.48
	Cov1	Age									
	ADD	Additive model									
	DOMDEV	Dominant model									

Abbreviations and keys: COPD-S: Patients with COPD related to tobacco smoking, CHR: Chromosome, SNP: Single Nucleotide Polymorphism, BP: Base pair location, A1: minor allele for each SNP, NMISS: No-missing data, OR: Odds ratio, SE: Standard error, L95: Lower interval confidence, U95: Upper interval confidence, ADD: Additive effect model, DOMDEV: Dominance deviation, COV1-4: Covariables. GENO_2DF: Genotypic 2 Degree-freedom.

Supplementary Table 3. Logistic regression analysis by co-variables and alleles in the COPD-BB group.

COPD-BB comparison (Alleles)											
CHR	SNP	BP	A1	TEST	NMISS	OR	SE	L95	U95	STAT	p-value
6	rs8193036	52185695	2	ADD	354	0.9822	0.2233	0.634	1.521	-0.08063	0.9357
6	rs8193036	52185695	2	SEX	354	18.94	0.8257	3.754	95.54	3.562	**0.0003678**
6	rs8193036	52185695	2	COV1	354	1.11	0.01242	1.083	1.137	8.399	**4.52E-17**
6	rs2275913	52186235	1	ADD	352	1.368	0.2653	0.8131	2.301	1.18	0.2379
6	rs2275913	52186235	1	SEX	352	17.78	0.8276	3.511	90.02	3.477	**0.0005062**
6	rs2275913	52186235	1	COV1	352	1.111	0.01245	1.084	1.138	8.441	**3.15E-17**
	cov1	Age									

Exacerbations among COPD-BB (Alleles)

CHR	SNP	BP	A1	TEST	NMISS	OR	SE	L95	U95	STAT	p-value
6	rs8193036	52185695	2	COV1	132	0.9576	0.02145	0.9182	0.9987	-2.019	**0.04346**
6	rs2275913	52186235	1	COV1	132	0.9556	0.02151	0.9161	0.9967	-2.113	**0.03463**

cov1 Age

Abbreviations and keys: COPD-BB: Patients with COPD related to biomass burning, CHR: Chromosome, SNP: Single Nucleotide Polymorphism, BP: Base pair location, A1: minor allele for each SNP, NMISS: No-missing data, OR: Odds ratio, SE: Standard error, L95: Lower interval confidence, U95: Upper interval confidence, ADD: Additive effect model, COV1-4: Covariables.

Supplementary Table 4. Logistic regression analysis by co-variables and genotypes in the COPD-BB group.

COPD-BB comparison (Genotypes)											
CHR	SNP	BP	A1	TEST	NMISS	OR	SE	L95	U95	STAT	P
6	rs8193036	52185695	2	ADD	354	0.9228	0.3329	0.4806	1.772	-0.2413	0.8093
6	rs8193036	52185695	2	DOMDEV	354	1.074	0.392	0.4979	2.315	0.181	0.8564
6	rs8193036	52185695	2	COV1	354	1.103	0.01177	1.078	1.129	8.339	**7.46E-17**
6	rs8193036	52185695	2	GENO_2DF	354	NA	NA	NA	NA	0.05823	0.9713
6	rs2275913	52186235	1	ADD	352	0.8284	0.6464	0.2334	2.941	-0.2913	0.7708
6	rs2275913	52186235	1	DOMDEV	352	1.94	0.6882	0.5036	7.476	0.9631	0.3355
6	rs2275913	52186235	1	COV1	352	1.104	0.01187	1.079	1.13	8.374	**5.55E-17**
6	rs2275913	52186235	1	GENO_2DF	352	NA	NA	NA	NA	3.009	0.2222

Exacerbations among COPD-BB (Genotypes)											
CHR	SNP	BP	A1	TEST	NMISS	OR	SE	L95	U95	STAT	P
6	rs8193036	52185695	2	ADD	132	3.75E-05	5423	0	inf	-0.00188	0.9985
6	rs8193036	52185695	2	DOMDEV	132	6.17E+04	5423	0	inf	0.002034	0.9984
6	rs8193036	52185695	2	COV1	132	0.9591	0.02188	0.9188	1.001	-1.91	0.05615
6	rs8193036	52185695	2	GENO_2DF	132	NA	NA	NA	NA	4.807	0.09042
6	rs2275913	52186235	1	ADD	132	NA	NA	NA	NA	NA	NA
6	rs2275913	52186235	1	DOMDEV	132	NA	NA	NA	NA	NA	NA
6	rs2275913	52186235	1	COV1	132	NA	NA	NA	NA	NA	NA
6	rs2275913	52186235	1	GENO_2DF	132	NA	NA	NA	NA	NA	NA

Abbreviations and keys: COPD-S: Patients with COPD related to biomass burning, CHR: Chromosome, SNP: Single Nucleotide Polymorphism, BP: Base pair location, A1: minor allele for each SNP, NMISS: No-missing data, OR: Odds ratio, SE: Standard error, L95: Lower interval confidence, U95: Upper interval confidence, ADD: Additive effect model, DOMDEV: Dominance deviation, COV1-4: Covariables. GENO_2DF: Genotypic 2 Degree-freedom.

Supplementary figures

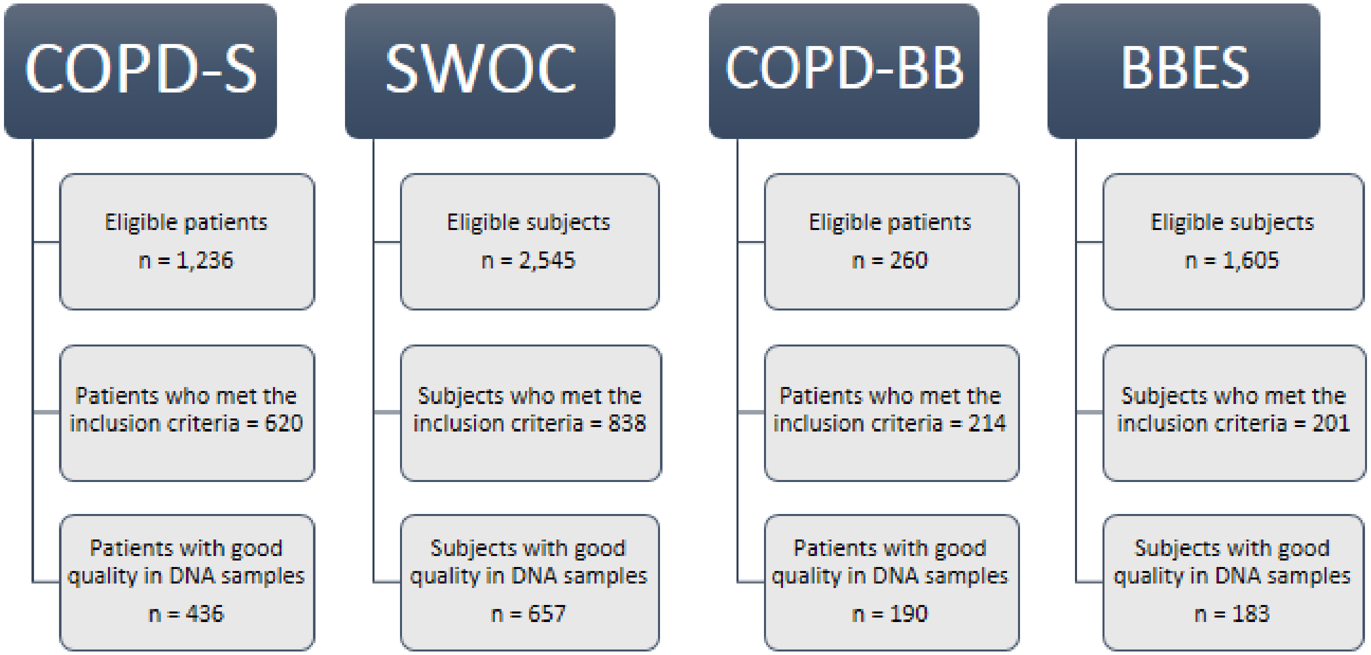

Supplementary figure 1. Eligible and enrolled subjects in the study.

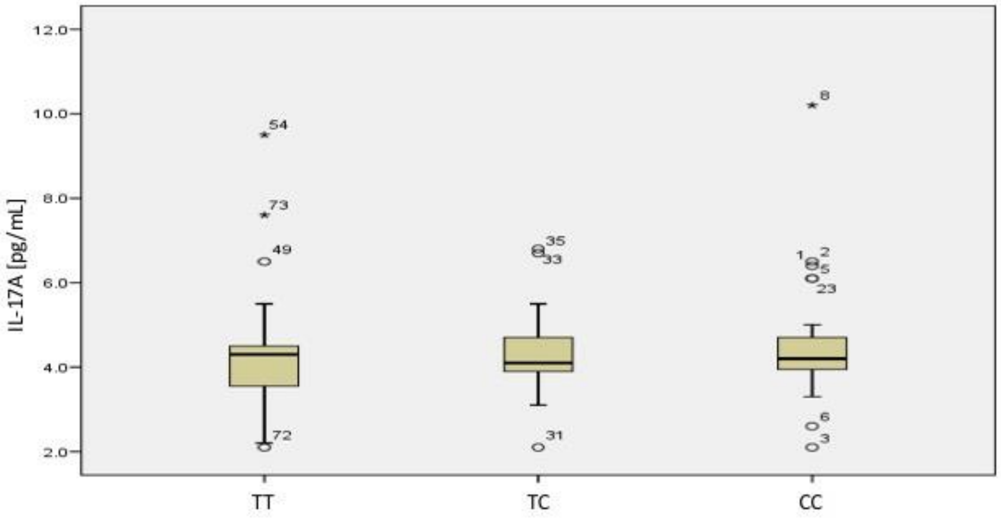

Supplementary Figure 2. IL-17A serum levels between genotypes of rs8193036 in COPD-S group.
